# Physical and Physiological Characteristics of Judo Athletes: An Update

**DOI:** 10.3390/sports4010020

**Published:** 2016-03-10

**Authors:** Gema Torres-Luque, Raquel Hernández-García, Raquel Escobar-Molina, Nuria Garatachea, Pantelis T. Nikolaidis

**Affiliations:** 1 Faculty of Humanities and Science Education, University of Jaen, Jaen 23071, Spain; 2 Faculty of Sport Science, University of Murcia, Murcia 30720, Spain; raquelhgarcia@gmail.com; 3 Faculty of Sport Science, University of Granada, Granada 18001, Spain; rescobar@ugr.es; 4 Faculty of Health and Sport Science, University of Zaragoza, Zaragoza 22002, Spain; nugarata@unizar.es; 5 Department of Physical and Cultural Education, Hellenic Army Academy, Athens 16673, Greece; pademil@hotmail.com; 6 Exercise Physiology Laboratory, Nikaia 18450, Greece

**Keywords:** anthropometry, evaluation, judo, physiology, training

## Abstract

Judo competition is characterized structurally by weight category, which raises the importance of physiological control training in judo. The aim of the present review was to examine scientific papers on the physiological profile of the judokas, maintenance or loss of weight, framing issues, such as anthropometric parameters (body fat percentage), heart rate responses to training and combat, maximal oxygen uptake, hematological, biological and hormones indicators. The values shown in this review should be used as a reference for the evaluation of physical fitness and the effectiveness of training programs. Hence, this information is expected to contribute to the development of optimal training interventions aiming to achieve maximum athletic performance and to maintain the health of judokas.

## 1. Introduction

Judo became an official sport event in the Olympic Games of Munich in 1972 for men and in the 1992 Games (Barcelona) for women (International Judo Federation). In the 1964 Games, judo was invited in Tokyo for men, whereas the same happened for women in the 1988 Games (Seoul). In 1961, there were world championships in Paris. The first competition with weight categories took place in the 1964 Games (Tokyo), including three categories and an open, while in 1965 (Rio de Janeiro), five categories and an open were included in the program.

Competitive judo demands high-intensity intermittent actions, in which optimal physical attributes are necessary in order to achieve technical-tactical development and success in combat [1,2]. Actually, high training loads, which require successful and coordinated actions, are applied to judokas in order to achieve high sport’s performance. Since judo is a sport including weight categories [3], a major training goal is the achievement of an optimal weight through the combination of restricted diet and aerobic training, usually aiming to decrease fat mass rapidly [4]. According to several studies, there are judokas who can lose between 3% and 6% fat mass during the last weeks before the competition [5,6,7,8,9,10]. This decrease might reach even 10% of body mass [11] in an attempt to get an advantage by competing against lighter, smaller and weaker opponents [12].

Judo trainers and judokas should have a solid knowledge about physiological responses to competitions and physiological adaptations to training in order to design an adequate training session and season [13]. A combination of high demands of training and rapid weight loss before competition could induce muscle damage and increase the risk of damage in other tissue [14]. The cumulative effect of weight changes along a complete season together with intense training load could be even more harmful and put judokas’ health at risk.

There are many authors who have written about the variation of physical and physiological profile of judokas during the training season [9,15], as well as other studies that have monitored this profile in the competitive time [5,7,16]. One of the most studied aspects is the effects of rapid weight loss on performance, where it has been reported that even in the case of “weight cycling” (down periodically), the body seems to suffer from physiological adaptation to these cycles [12]. For example, senior judokas presented higher use of weight loss methods, especially in the period one week before competitions compared to their junior counterparts. Judokas were involved in their diets and reduced more weight to a greater extent as they were getting older [17]. This review aimed to bring together all of those studies that have framed the physical and physiological characteristics of judokas.

## 2. Body Composition

Judo is a combat sport with strict competitive weight categories. In 1964, when judo became an official Olympic event, a competitive weight class system was adopted. Judokas should optimally select the weight class that is appropriate to their height and physique. However, many of them often undergo severe weight reduction through calorie restriction in order to select a lower-weight class and, in this way, to gain an advantage over other judokas in a particular weight class. In order to achieve the weight that would allow them to participate in a specific class, many contestants undergo drastic food restriction, especially during the week preceding the competition. The amount of weight loss is subsequently regained as the athletes compensate for the sustained energy drain by excess food intake during the post-competition period [18]. This rapid alternation between weight loss and regain is known as “weight cycling”. Artioli and his colleagues [3] highlighted the need to assess the importance of maintaining weight in judo after learning that 80% of judokas performed fast weight descents. Ebine and colleagues have described anthropometric characteristics and weight in high-level judokas distinguished by sex and weight category [19].

Body mass and body fat percentage are essential measurements that could change largely according to sex, age, weight category and training. With regards to sex, it has been established that body fat percentage for elite female judokas is about 10% higher than for male judokas [20]. A study indicated that male judokas were heavier, taller, had lower body fat and higher percentage and absolute values of muscle mass, higher circumferences and bone diameters, lower endomorphic and higher mesomorphic components than females [21].

With regards to age, it has been noticed that in young judokas (14–18 years old), the body fat percentage is between 10% and 16%, whereas in older judokas (19–24 years old), it was 8%–10% in the case of the international level and 8%–15% in the case of the national level [22]. Female judokas of 15–16 years old presented body fat percentage from 16%–23% [23], and female college judokas aged 19–20 years old had 19.3% [24]. These data were very close those found by Franchini *et al.* [21] in the assessment of the Spanish judo team from cadets to seniors. The latter authors reported values as follows: in females, cadet category (16 years old), 19.5% body fat, junior category (20 years old), 24.0% body fat and in senior category (22 years old), 19.9%; in males, of the cadet category, 12.1% body fat, in the junior category, 10.6% body fat, and the senior category, 8% body fat; as for the values of muscle mass, in females, 44%, 41.5% and 44.7% in the cadet, junior and senior category, respectively; in males, 48.5%, 50.3% and 53.2% of muscle body mass.

Various studies have compared body fat percentage among groups differing for competitive level (e.g., international *vs.* national level). It was well documented that national-level judokas had a higher body fat percentage than international level [4,20,25]. A study [26] demonstrated that judokas who participated in the Olympic Games or Asian Games (international level) had significantly higher fat-free mass than university judokas (university level) who did not participate in intercollegiate competitions. In fact, the muscle density normalized to the height of the individual was larger in judokas at the international level than in those at the university level. World- and Olympic-level male judokas usually had lower than 10% body fat [16]. Nevertheless, caution was needed when using this value as a reference, because most studies predicted body fat by skinfold thickness measurements, and therefore, the specific mean error of the estimate of each equation should be taken into consideration [21]. To sum up, the abovementioned studies showed that body fat percentage decreased as competition level increased.

With regards to the association between competition level and body fat percentage, an alternative research approach to the comparison between groups differing for competition level was to examine correlations. These fat mass differences between competitive levels had an important repercussion in sport’s performance [16]. Franchini *et al.* [16] compared the morphological characteristics of the male judokas of the Brazilian Team A with the judokas of Teams B and C (reserves). These authors concluded that a higher body fat percentage was negatively correlated with performance in activities with body mass locomotion. However, the aforementioned correlation was not statistically significant in adolescent judokas [27].

Another study found that judokas in the heaviest weight categories were stronger (relative to lean body mass values) than those in the lightest categories [28]. Some other studies compared weight categories concerning the body fat percentage, and they found a linear increase from the under-60 kg to the 81–90 kg category and a large increase in the half-heavy-weight (100 kg) and heavy-weight (more than 100 kg) categories [28].

Because judo is a weight-classified sport, the body mass changes along the sport, season especially when judokas are preparing for a competition. A decrease of body fat percentage (3%–6%) during the last weeks before competition has been described [5,6,7,8,9,10,29] and even 10% in another case [11]. It was recommended to assess body composition in different periods of the season, at least on three or four occasions [7,8,9,29,30]. Several studies reported that fat mass percentage during the competition period was lower than during rest or pre-competition periods [7,27,29,31]. For this reason, Artioli *et al.* [3] proposed six basic rules for the control of the judoka weight and the prevention of the negative effects of the sudden loss of weight in this sport’s performance: (1) the combats begin no more than 1 h after the weigh-in; (2) each athlete is allowed to weigh-in only on time; (3) rapid weight loss methods and artificial rehydration methods are prohibited in the days of the competition; (4) athletes must pass the hydration test to get the weigh-in validated; (5) an individual minimum competitive weight is determined at the beginning of each season; and (6) no athletes are allowed to compete in a weight class that would require weight loss greater than 1.5% of body weight per week. Moreover, the fat mass percentage of high level judokas was usually lower than the sedentary population [32] (Table 1 and Table 2).

## 3. Physical Fitness

Maximal oxygen uptake (VO_2_max) has been used as a measure of aerobic capacity in male and female judokas. The literature has reported VO_2_max values between 43 and 65 mL·kg^−1^·min^−1^ [54] (Table 3). Male judokas had higher VO_2_max values (from 44–64 mL·kg^−1^·min^−1^) than females (from 43–53 mL·kg^−1^·min^−1^) [4,15,28,55]. Considering VO_2_max by weight categories, values from 39–57 mL·kg^−1^·min^−1^ were reported for lightweight, from 42–59.5 mL·kg^−1^·min^−1^ for medium weight and from 42–59.5 mL·kg^−1^·min^−1^ for the heavy weight category [4,15,28,55].

Although the aerobic profile of judokas has been well documented by the aforementioned studies, the chronic adaptations of aerobic capacity to judo training were less studied [4,28,56]. There was a study supporting that judo training might induce an increase of VO_2_max, where De Cree *et al.* [4] reported a significant increase of VO_2_max after six weeks of judo training in woman around 17–29 years. In contrast, Callister *et al.* [28] did not find changes in VO_2_max after 10 weeks in elite judokas. An interpretation for the lack of changes in the latter study might be the level of judokas; since they were elite, they would be expected to possess a high level of VO_2_max at baseline, which would be difficult to improve further. On the other side, such a lack of changes might be due to the nature of judo training, which is more anaerobic rather than aerobic and does not consist of an exercise stimulus for improvement of the high level of VO_2_max. Thus, it is necessary to conduct more research on the effects of judo training on VO_2_max. Franchini *et al.* [56] showed the values of VO_2_ in males judokas of 25 years old while performing nine intermittent series of uchikomis for one minute: VO_2_ differed over time during activity with lower values during the first minute (32 mL·kg^−1^·min^−1^).

In addition to the abovementioned research that focused on aerobic capacity, several studies have profiled other parameters of physical fitness, such as flexibility, handgrip muscle strength and vertical jump of judokas [33,35,36,40,47,51,52] (Table 4, Table 5 and Table 6). The data presented by these studies provided valuable reference values of physical fitness in other judo practitioners (e.g., coaches, fitness trainers) to evaluate and monitor their judokas.

## 4. Heart Rate

The assessment of heart rate (HR) at rest (HR_rest_ submaximal and maximal intensities (HR_max_) contributes to monitoring of aerobic capacity and exercise intensity. HRrest in male judokas ranged from 54–65 bpm and was slightly lower than in female judokas (65–71 bpm) [15,19,55]. During judo contests, several studies have reported mean HR in male judokas of 180–182 bpm, which corresponded to 85%–90% of HR_max_ [15,55,57,58]. HRmax during judo contests was 190–200 bpm in male and slightly lower in female judokas [4,58].

HR has been also used to quantify exercise intensity during judo training. For instance, a study [59] classified the intensity of the different efforts of judo training. The most common efforts of judo training were put in order from the highest to lowest intensity as follows: *randori* (4 min and 30 s), *randori* (5 min 30 s), *uchi-komi* once each every 3 s, *nage-komi* every 3 s during 1 min 30 s, *uchi-komi* once each every 4 s and *nage-komi* every 4 s during 1 min 30 s. Moreover, Franchini *et al.* [56] in a more recent study evaluated the performance and physiological responses in intermittent training uchikomi and showed HR scores during execution of 183 bpm and during rest of 95 bpm, with lower values observed in the first minute compared to the second and third minute.

## 5. Blood Lactate

The concentration of lactate in blood [La^−^] has been extensively studied in judo, both in real competition, simulations of competition and training. [La^−^] values have ranged 7–10 mmol·L^−1^ after combat simulations [15,55,58,60]. In simulated judo contests, differences in [La^−^] have not been found, either between competition levels [2] or sexes [58]. Callister *et al.* [28] reported [La^−^] of 9.1 ± 1.1 mmol·L^−1^ after a specific training judo (*uchikomis*, *ne waza* and 3–7 *randori*). In addition, Franchini *et al.* [61] found significant increments after a simulated contest. In a recent review [56], there were variations in [La^−^] after combats, at least in simulated combat. In these studies, it was found that peak [La^−^] was higher after the first combat compared to the second, third and fourth combat, about 2 mmol·L^−1^ [22,62]. Taken together, these studies suggested a decrease in anaerobic lactic contribution as the judokas performed consecutive combats [56]. Serrano *et al.* [60] found a positive correlation between the rate of perceived exertion and maximal [La^−^] during competition in regional-level male judokas.

## 6. Hematological Evaluation

Hematological evaluation in judokas has focused on concentrations of total erythrocytes and leukocytes, as well as quantification of hemoglobin (Hb) and hematocrit (Hct). Malczewska *et al.* [63] reported total erythrocyte and leukocyte levels in male high level judokas of 4.82 × 10^12^ L^−1^ and 5.8 × 10^9^ L^−1^, respectively. These authors reported values of Hb of 15.5 g·L^−1^ and Hct of 43% for the same group of judokas. In female high level judokas, Hb was 12.5 g·dL^−1^ and 38% for Hct [64]. In the case of young judokas (16 years old), Hb and Hct have been reported a bit lower (137 g·L^−1^ and 42%, respectively) than in adult judokas [65].

Hb and Hct did not change significantly during a 20-day rapid weight loss period before competition [7,29]. A study [66] has found similar results in junior male judokas; in this study, Hb and Htc did not change after training. In contrast, Su *et al.* [65] found a significant decrement in Hb (from 137 down to 128 g·L^−1^) and Htc (from 42% down to 39%) after a five-week training period in young judokas (16 years old). This finding was in agreement with that of Umeda *et al.* [24], who showed that the effect of a training round for seven days resulted in a significant decrease in leukocytes and Hb levels in a group of female judokas (19 years old). Furthermore, another study investigated the combined effects of dietary restriction and weight reduction through exercise on markers of immune function in college judokas before and after a single competition [29]. These authors found that the weight reduction group exhibited significant decreases in concentrations of serum immunoglobulins and complements (IgG, IgM and C3) at seven days after the competition. In the dietary restriction group, significant decreases in IgM and C3 were observed at seven days after the competition, though to a lesser degree than in the weight reduction group. Moreover, the authors of this study observed a significant decrease in total leukocytes one day before competition (from 6329 down to 5497 cel·mm^2^). This study concluded that energy restriction seemed to exacerbate alterations in immune markers, such as immunoglobulin, and complements induced by vigorous exercise at seven days after a competition. Although the changed values were still within normal limits, the authors hypothesized that the potential cumulative effect of these changes over many competitions in one year might well induce abnormal levels with a possibly harmful clinical effect on judokas. In contrast, the study of Kowatari *et al.* [5] failed to find changes in leukocytes, lymphocytes and neutrophils after a combined weight reduction and training program.

On the other hand, white blood cells and neutrophils in the blood values showed a significant increase after training judo, ±1.2–10.7 ± 1.8 × 10^3^/µL and 2.98 ± 0.82–7.95 ±1.8 × 10^3^/µL, respectively, in 18 male judokas [66]. Another study evaluating the effects of an intense training camp of seven days in female judokas of 19 years old showed a decreased in immunoglobulin after training compared with baseline values, suggesting the appearance and accumulation of physical fatigue [24].

Ohta *et al.* [7] and Umeda *et al.* [29] controlled IgG, IgA and IgM variability four times (Days 20 and 7, one day before the competition and five days later) in judokas depending on the range of weight loss. Ohta *et al.* [7] showed a significant reduction in the three types of immunoglobulins in the group with a high reduction of weight; however, in Umeda *et al.* [29] only was a decrease in IgG and IgM in the group of normal weight reduction observed. Concerning weight maintenance, group values remain constant in both works during the period of the study; coinciding with some of the above authors claiming that a sharp reduction of body weight along with the practice of intense training may induce a decreased ability of the immune system of athletes [7,29].

Kim *et al.* [32] pointed out that studies focused on the immune system of female judokas are limited. They found that the level of immunoglobulins (IgG and IgM) in female judokas was lower than in sedentary females. These results reinforced the fact that high demands of training linked with imbalanced nutrition in female judokas could induce a low immune function and impairments in sport’s performance and health status.

## 7. Free Fatty Acids and Glycerol

Among sports where athletes are categorized by weight, judo is characterized by relatively short duration, high intensity and intermittent exercise with a combat lasting ~7.18 min in males. It has been shown that a single judo combat is able to induce mobilization of both protein and lipid metabolism [55].

Finaud *et al.* [8] observed in national judokas that after a week of weight loss, the triglyceride concentration decreases significantly compared to the initial values, and also, triglycerides in the blood after the competition levels are lower than before they compete. However, free fatty acids, after having increased significantly after a week of reduction of body weight, return to the initial values after the competition. With respect to glycerol, a significant increase in blood concentration occurs the day before a competition, and then continues after the championship, but not significantly. In the control group, only statistically significant differences in the levels of glycerol appear after the competition. These results were similar to other studies [7,30].

On the contrary, Filaire *et al.* [67] observed a significant increase in the concentration of triglycerides in blood after a week of male judokas of −73 kg on food restriction. With regards to glycerol concentrations, they decreased after a week of strict diet, but without reaching statistically significant differences (0.12–0.17 mmol·L^−1^).

A study [15] about the contribution of protein metabolism during judo combat and recovery showed significant increments in triglycerides, free fatty acids and glycerol 3 min after judo combat; similarly, there was a significant increase in free fatty acids and glycerol 60 min after combat. Glycerol levels obtained 3 and 60 min after the end of the combat are above the rank of normality (50–100 µmol·L^−1^). Therefore, the authors hypothesize that since the judoka study was carried out with the misleading use of lipid metabolism (lipolysis), bringing with it the increase in free fatty acids and glycerol after the effort, characterized by an intake of a carbohydrate diet rather than that recommended, this can be the case. 

## 8. Testosterone and Cortisol

Stress management before a competition for athletes is very important, especially for those who reduce their weight, such as judokas. In general, adrenocortical hormone is well known as a stress marker, and cortisol (C) is representative of it. Nowadays, the ratio of testosterone (T) to C (T/C) is a good marker of overtraining diagnosis [68]. In a study aiming to investigate the salivary T and C and the mental state responses to a real judo championship [69], C response to competition was shown, which was especially characterized by an anticipatory rise, which was confirmed later by Salvador *et al.* [70] and Obminski [71], suggesting that increased levels of C on judokas’ competition day predisposed them to success. Depending on the outcome, the results did not show significant differences in C responses. T values recorded after the last fight were significantly greater in the losers than those in the winners. Hormonal response did not show a relationship with psychological variables depending on the outcome. Finally, even if no relationship between hormonal and psychological variables depending on the outcome was found, the results showed that state and trait psychological variables, as well as the coping strategies, must be taken into account to better understand the response to competitive situations.

Salvador *et al.* [70] compared the anticipatory hormonal and psychological responses of 17 male judokas in the resting sessions and before competition sessions along the official competition. C levels and anxiety scores were concurrently higher before the contest than in resting conditions. Their results showed that one group of subjects did display T increases, higher C levels and higher motivation to win scores than the other group, indicating that this hormonal pattern and its relationships with psychological variables suggested an adaptive psychobiological response to a competition.

The relationships between psychophysiological variables have been investigated at two levels (regional *vs.* interregional) by comparing physiological responses (salivary C and T concentrations) and psychological responses prior to judo competitions [69]. C levels increased sharply (about 2.5-fold resting levels) throughout both competitions with no changes in T levels. Positive relationships between anxiety components (somatic and cognitive anxiety) and C were noted in both competitions. The study concluded that salivary C, together with anxiety components, may provide a better sensitivity index of physiological stress than T concentrations.

Correlations between T levels and fighting in male participants in judo contests have been studied [72]. A positive relation between T and offensive behaviors was obtained in the sense that the greater the hormonal titer, the greater the number of threats, fights and attacks. These findings coincide with the pattern of relationships found using observational scales. Conversely, C also presented positive correlations with some of these behavioral categories, but did not moderate the relationship between T and competitive behavior. These results give support to the view that T can be linked to the expression of competitive aggression in judo.

Toda *et al.* [6] investigated the effect of weight reduction prior to a competition on the salivary C level of judokas varying for the reduction rate (WRR, %). In this study, participants were divided into three groups depending on their WRR: the no-weight reduction group (WRR < 3%), the low-weight reduction group (3 ≤ WRR < 6%) and the high-weight reduction group. The collection of the measurements was carried out on three different days: 20 days before the competition (before weight reduction), the day before the competition (the peak of weight reduction) and seven days after the competition. There was no significant change in the salivary C levels during the weight reduction. On the day before the competition, however, those of the low- and high-weight reduction groups showed a tendency to decrease and were significantly lower than that of the no-weight reduction group. In addition, their recovery was observed at seven days after the competition. These findings suggested that the HPA axis is affected during the relatively early stage of weight reduction.

Degoutte *et al.* [30] examined the effects of weight loss induced by restricting energy and fluid intake on the physiology, psychology and physical performance of judo athletes. These authors observed that the plasma level of C, after a significant weight reduction, increased significantly, whereas the T and T/C ratio decreased significantly. These results suggested that the combination of energy restriction and intense exercise training, resulting in weight reduction before a competition, adversely affected the physiology and psychology of judo athletes and impaired physical performance before the competition. The results of another study [9], in line with those of Degoutte *et al.* [30], showed an 81% increment in C level compared to the baseline value, although when the normal weight was recovered again, the C decreased 27%, indicating that catabolic processes were augmented after weight reduction.

This study has a something’s limitations. It discussed a compendium of studies related to the functional and physiological characteristics judo athlete. However, it is not a systematic review of these aspects. Coaches can consider directions and guidelines; and can serve as support for their workouts. Therefore, data must be taken with caution.

## 9. Conclusions

The values shown in this review are vital for training control. It collaborates with the planning of training and maintaining the health of judokas.

## Figures and Tables

**Table 1:** Body mass, height and body mass index in judokas.

Sample	Age (years)	Body Mass (kg)	Height (cm)	BMI (kg·m^−2^)	Reference
Males					
Spanish (*n* = 74)	14.7 ± 1.1	60.8 ± 13.8	168 ± 10	21.4 ± 3.0	(Torres-Luque *et al.*, 2015) [33]
Koreans (*n* = 28)	16.0 ± 0.9	67.0 ± 9.7	175 ± 6	21.9 ^a^	(Kim *et al.*, 2011) [34]
French national (*n* = 10)	17 ± 1	71.4			(Koral and Dosseville, 2009) [35]
Tunisians national (*n* = 15)	18 ± 1	68.1 ± 8	1.74 ^a^	22.4 ± 1.8	(Chaouachi *et al.*, 2009) [36]
Tunisians regional (*n* = 10)	18.1 ± 1.7	77.2 ± 11.7	176 ± 5	24.9 ^a^	(El Abed *et al.*, 2009) [37]
Tunisians national (*n* = 12)	18.6 ± 2.4	77.1 ± 10.7	178 ± 6	24.3 ^a^	(Souissi *et al.*, 2013) [38]
Japans collegiate (*n* = 14)	19.9 ± 1.1	68.9 ± 5.0	169 ± 3	24.1 ^a^	(Iwai *et al.*, 2008) [39]
Cypriots competitive (*n* = 11)	20 ± 6	74.9 ± 12.1	172 ± 4	25.3 ^a^	(Papacosta *et al.*, 2013) [40]
Koreans collegiate (*n* = 26)	20.4 ± 0.7	78.6 ± 15.2	174 ± 7	26.0 ^a^	(Kim *et al.*, 2011) [34]
Brazilian professional (*n* = 39)	20.4	73.7	170	25.5	(Saraiva *et al.*, 2014) [41]
Brazilian national (*n* = 20)	20.7 ± 4.6	72.8 ± 12.6	174 ± 9	24.0 ^a^	(Detanico *et al.*, 2015) [42]
Spanish national (*n* = 12)	22.0 ± 3.2	76.3 ± 12.7	176 ± 7	24.6 ^a^	(Bonitch-Góngora *et al.*, 2012) [43]
Polish competitive (n = 22)	22.2 ± 3.6	87.5 ± 24.9	180 ± 8	26.8 ± 5.2	(Sterkowicz-Przybycień and Almansba, 2011) [44]
Brazilian competitive (*n* = 22)	22.5 ± 3.9	76.9 ± 9.1	176 ± 5	24.8 ^a^	(Ache Dias *et al.*, 2012) [45]
French national (*n* = 10)	22.6 ± 2.1	85.8 ± 10.2	180 ± 7	26.5 ^a^	(Cottin *et al.*, 2001) [46]
Spanish national (*n* = 14)	22.9	76.9	174	25.4 ^a^	(Morales *et al.*, 2014) [47]
Portuguese national (*n* = 27)	23.2 ± 2.8	72.8 ± 7.1	176 ± 5	23.6 ± 2.3	(Silva *et al.*, 2010) [48] (Silva *et al.*, 2011) [49]
Portuguese national (*n* = 32)	23.2 ± 3.3	73.4 ± 8.3	174 ± 5	24.2 ^a^	(Gonçalves *et al.*, 2015) [50]
Korean national (*n* = 10)	24.3 ± 3.3	81.4 ± 14.3	174 ± 8	26.9 ^a^	(Kim *et al.*, 2011) [34]
Europeans elite (*n* = 10)	25.6	100.7	186	29.1 ^a^	(Drid *et al.*, 2015) [51]
Brazilian recreational (*n* = 180)	25.7 ± 4.9	78.3 ± 15.1	~174	25.9 ± 4.0	(Schwart *et al.*, 2015) [52]
French competitive (*n* = 13)	adults	78.7	173	26.3 ^a^	(Franchini *et al.*, 2015) [53]
Females					
Spanish (*n* = 72)	14.5 ± 1.2	56.4 ± 13.1	162 ± 7	21.4 ± 4.2	(Torres-Luque *et al.*, 2015) [33]
French national (*n* = 10)	17 ± 1	69.6			(Koral and Dosseville, 2009) [35]
Polish competitive (*n* = 12)	23.1 ± 1.7	74.9 ± 24.0	169 ± 8	25.7 ± 5.6	(Sterkowicz-Przybycień and Almansba, 2011) [44]

BMI = body mass index. ^a^ Calculated by the authors of the present study.

**Table 2:** Body composition in judokas.

Sample	Age (years)	BF (%)	Assessment Method	Reference
Males				
Spanish (*n* = 74)	14.7 ± 1.1	12.7 ± 4.5	BIA	(Torres-Luque *et al.*, 2015) [33]
Koreans (*n* = 28)	16.0 ± 0.9	12.9 ± 2.8	BIA	(Kim *et al.*, 2011) [34]
French national (*n* = 10)	17 ± 1	12.7	4 skinfolds	(Koral and Dosseville, 2009) [35]
Cypriots competitive (*n* = 11)	20 ± 6	8.1 ± 1.9	skinfolds	(Papacosta *et al.*, 2013) [40]
Koreans collegiate (*n* = 26)	20.4 ± 0.7	14.1 ± 2.5	BIA	(Kim *et al.*, 2011) [34]
Brazilian national (*n* = 20)	20.7 ± 4.6	13.9 ± 3.1	–	(Detanico *et al.*, 2015) [42]
Spanish national (*n* = 12)	22.0 ± 3.2	15.3	skinfolds	(Bonitch-Góngora *et al.*, 2012) [43]
Polish competitive (*n* = 22)	22.2 ± 3.6	14.3 ± 4.3	2 skinfolds	(Sterkowicz-Przybycień and Almansba, 2011) [44]
Portuguese national (*n* = 27)	23.2 ± 2.8	12.1 ± 3.1	DEXA	(Silva *et al.*, 2010, 2011) [48,49]
Portuguese national (*n* = 32)	23.2 ± 3.3	12.1	DEXA	(Gonçalves *et al.*, 2015) [50]
Korean national (*n* = 10)	24.3 ± 3.3	12.5 ± 3.1	BIA	(Kim *et al.*, 2011) [34]
Brazilian recreational (*n* = 180)	25.7 ± 4.9	15.7 ± 7.6	3 skinfolds	(Schwartz *et al.*, 2015) [52]
Females				
Spanish (*n* = 72)	14.5 ± 1.2	24.6 ± 7.5	BIA	(Torres-Luque *et al.*, 2015) [33]
French national (*n* = 10)	17 ± 1	23.1	4 skinfolds	(Koral and Dosseville, 2009) [35]
Polish competitive (*n* = 12)	23.1 ± 1.7	23.3 ± 3.7	2 skinfolds	(Sterkowicz-Przybycień and Almansba, 2011) [44]

BF = body fat percentage; BIA = bioelectrical impedance analysis; DEXA = dual-energy X-ray absorptiometry.

**Table 3:** Flexibility in judokas.

Sample	Age (years)	Flexibility (cm)	Assessment Method	Reference
Males				
Spanish (*n* = 74)	14.7 ± 1.1	2.0 ± 7.9	SAR	(Torres-Luque *et al.*, 2015) [33]
Brazilian recreational (*n* = 180)	25.7 ± 4.9	37.7 ± 11.9	SAR ^a^	(Schwartz *et al.*, 2015) [52]
Females				
Spanish (*n* = 72)	14.5 ± 1.2	4.9 ± 7.8	SAR	(Torres-Luque *et al.*, 2015) [33]

SAR = sit-and-reach test; ^a^ equipment provides an advantage of 26 cm.

**Table 4:** Handgrip muscle strength in judokas.

Sample	Age (years)	Right Hand (kg)	Left Hand (kg)	Sum of Two Hands (kg)	Reference
Males					
Spanish (*n* = 74)	14.7 ± 1.1	37.7 ± 11.9			(Torres-Luque *et al.*, 2015) [33]
Tunisians national (*n* = 12)	18.6 ± 2.4	~54			(Souissi *et al.*, 2013) [38]
Cypriots competitive (*n* = 11)	20 ± 6	46.5 ± 8.8	42.0 ± 8.8	88.5 ^a^	(Papacosta *et al.*, 2013) [40]
Spanish national (*n* = 12)	22.0 ± 3.2	57.6 ± 6.9	55.4 ± 7.4	113 ^a^	(Bonitch-Góngora *et al.*, 2012) [43]
Spanish national (*n* = 14)	22.9	48.4	46.5	94.9	(Morales *et al.*, 2014) [47]
Portuguese national (*n* = 27)	23.2 ± 2.8	50.8 ± 7.4 ^b^			(Silva *et al.*, 2011) [49]
Europeans elite (*n* = 10)	25.6 ± 3.6	64.3	69.0	133.3 ^a^	(Drid *et al.*, 2015) [51]
Brazilian recreational (*n* = 180)	25.7 ± 4.9			101 ± 15	(Schwartz *et al.*, 2015) [52]
Brazilian competitive (*n* = 22)	22.5 ± 3.9	51.4 ± 8.2 ^c^	51.4 ± 7.9	102.8 ^a^	(Ache Dias *et al.*, 2012) [45]
French competitive (*n* = 13)	adults	50.0	49.6	99.6 ^a^	(Franchini *et al.*, 2015) [53]
Females					
Spanish (*n* = 72)	14.5 ± 1.2	26.6 ± 7.4			(Torres-Luque *et al.*, 2015) [33]

^a^ Calculated by the authors of the present study; ^b^ dominant hand; ^c^ dominant and non-dominant hand.

**Table 5:** Vertical jump ability in judokas.

Sample	Age (years)	Jump (cm)	Assessment Method	Reference
Males				
Spanish (*n* = 74)	14.7 ± 1.1	30.8 ± 10.5	CMJ	(Torres-Luque *et al.*, 2015) [33]
French national (*n* = 10)	17 ± 1	57.3	SJ	(Koral and Dosseville, 2009) [35]
French national (*n* = 10)	17 ± 1	58.7	CMJ	(Koral and Dosseville, 2009) [35]
Tunisians national (*n* = 15)	18 ± 1	42.0 ± 3.2	SJ	(Chaouachi *et al.*, 2009) [36]
Tunisians national (*n* = 15)	18 ± 1	45.6 ± 4.2	CMJ	(Chaouachi *et al.*, 2009) [36]
Cypriots competitive (*n* = 11)	20 ± 6	41.7 ± 5.3	CMJ	(Papacosta *et al.*, 2013) [40]
Brazilian national (*n* = 20)	20.7 ± 4.6	45.4 ± 5.2	CMJ	(Detanico *et al.*, 2015) [42]
Females				
Spanish (*n* = 72)	14.5 ± 1.2	27.7 ± 13.5	CMJ	(Torres-Luque *et al.*, 2015) [33]
French national (*n* = 10)	17 ± 1	44.3	SJ	(Koral and Dosseville, 2009) [35]
French national (*n* = 10)	17 ± 1	45.3	CMJ	(Koral and Dosseville, 2009) [35]

CMJ = countermovement jump; SJ = squat jump.

**Table 6:** Aerobic capacity in judokas.

Sample	Age (years)	Aerobic Capacity	Assessment Method	Reference
Males				
Tunisians national (*n* = 15)	18 ± 1	53.3 ± 3.9	SRT	(Chaouachi *et al.*, 2009) [36]
Cypriots competitive (*n* = 11)	20 ± 6	57.2 ± 7.2	GXT ^b^	(Papacosta *et al.*, 2013) [40]
French national (*n* = 10)	22.6 ± 2.1	44.5 ± 6	GXT ^a^	(Cottin *et al.*, 2001) [46]
Europeans elite (*n* = 10)	25.6	56.0	GXT ^b^	(Drid *et al.*, 2015) [51]
Brazilian recreational (*n* = 180)	25.7 ± 4.9	52.2 ± 7.9	Queen’s College step test	(Schwartz *et al.*, 2015) [52]

SRT = 20-m shuttle run test; GXT = graded exercise test; ^a^ cycle ergometer; ^b^ treadmill.
